# The absence of the alpha-1 band in serum protein electrophoresis as a clue to diagnosis of alpha-1 antitrypsin deficiency panniculitis

**DOI:** 10.1016/j.jdcr.2026.01.004

**Published:** 2026-01-10

**Authors:** Erica Dale, Frank Z. Jing, Michael Camilleri, Kenneth J. Warrington, Afsaneh Alavi

**Affiliations:** a Mayo Clinic Alix School of Medicine, Rochester, Minnesota; b Department of Dermatology; Mayo Clinic, Rochester, Minnesota; c Division of Rheumatology; Mayo Clinic, Rochester, Minnesota

**Keywords:** alpha-1 antitrypsin, alpha-1 band, deficiency, panniculitis, serum electrophoresis, ulceration

## Case 1

**Fig 1:**
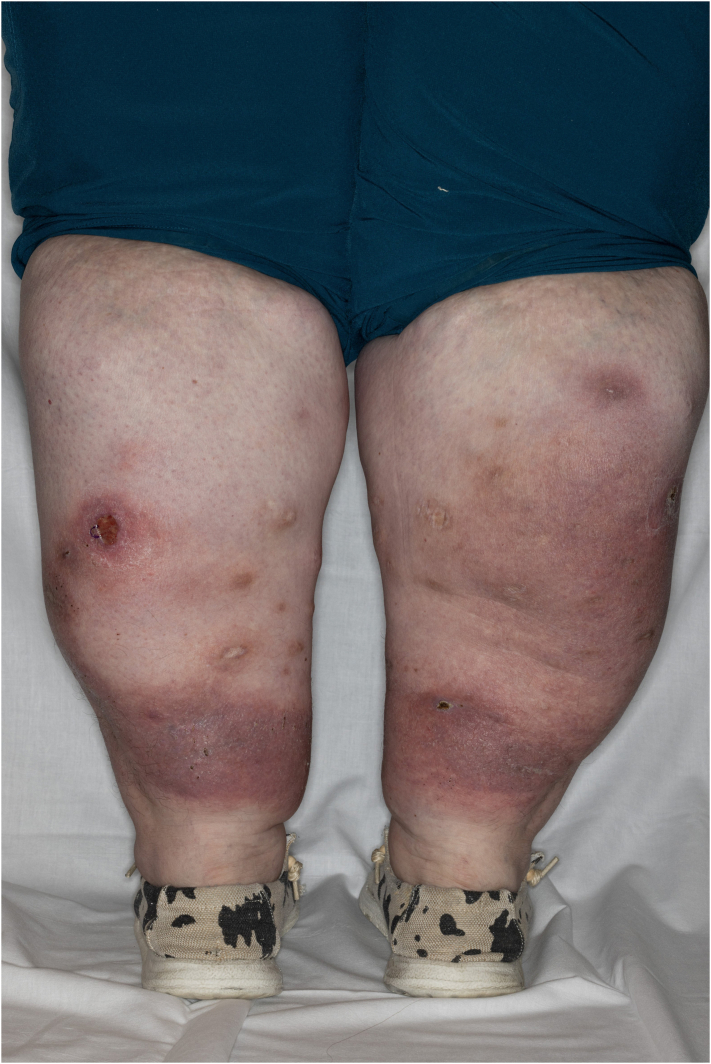
Clinical photograph of the posterior lower extremities.

**Fig 2:**
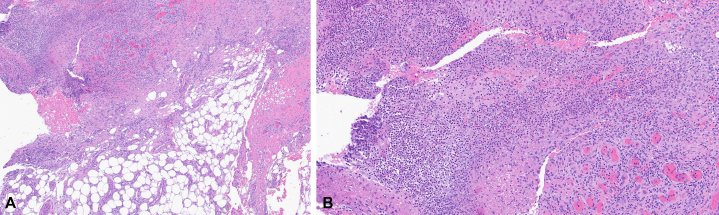
Hematoxylin and eosin stain of biopsy specimen obtained from the lower extremity. (Original magnifications: **A,** ×40; **B,** ×100).

A 39-year-old female with a medical history of morbid obesity and lipedema presented with multiple recurrent painful nodules and ulcerations on the lower extremities (Fig 1). The ulcerations began at age 30 and were often precipitated by minor trauma or friction. Initial punch skin biopsy was suggestive of lipodermatosclerosis. Laboratory studies revealed a markedly positive c-antineutrophil cytoplasmic antibody (c-ANCA) and elevated erythrocyte sedimentation rate. The patient was referred to rheumatology, and there was no clinical or imaging evidence of systemic small vessel vasculitis. Subsequent evaluation included venous and arterial studies that were unremarkable. An elliptical skin biopsy was performed, which revealed mid-to-deep dermal mixed neutrophilic infiltrate with dermal necrosis and hemorrhage and superficial dermal-to-superficial subcutaneous angiofibroplasia-fibrosis (Fig 2, *A* and *B*). Notably, a repeat c-ANCA showed continued positivity with a titer greater than 1:4096, while monoclonal gammopathy panel revealed absence of the alpha-1 band. Alpha-1-antitrypsin (AAT) phenotyping was conducted, revealing a significantly low level of 29 and a homozygous ZZ mutation. Given the new diagnosis of alpha-1-antitrypsin deficiency (AATD), the patient was referred to gastroenterology and pulmonology for further evaluation. She was started on doxycycline 100 mg daily for treatment of her panniculitis and was evaluated by pulmonology for alpha-1 antitrypsin replacement.

## Case 2

**Fig 3:**
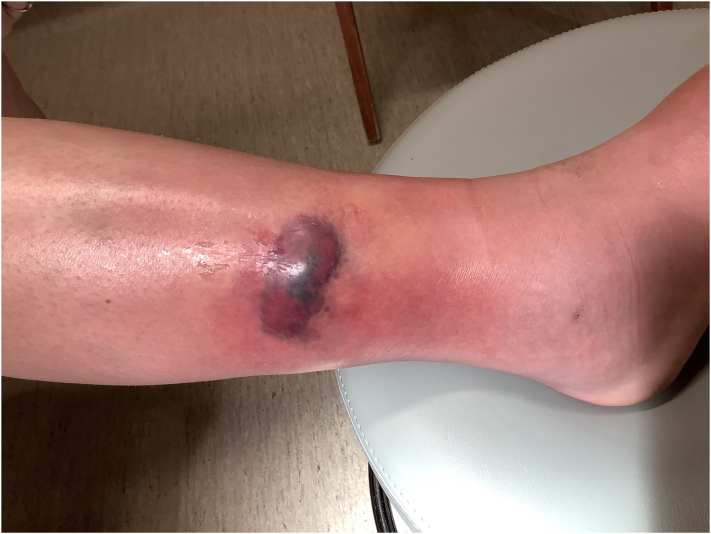
Clinical photograph of the right lateral lower extremity.

**Fig 4:**
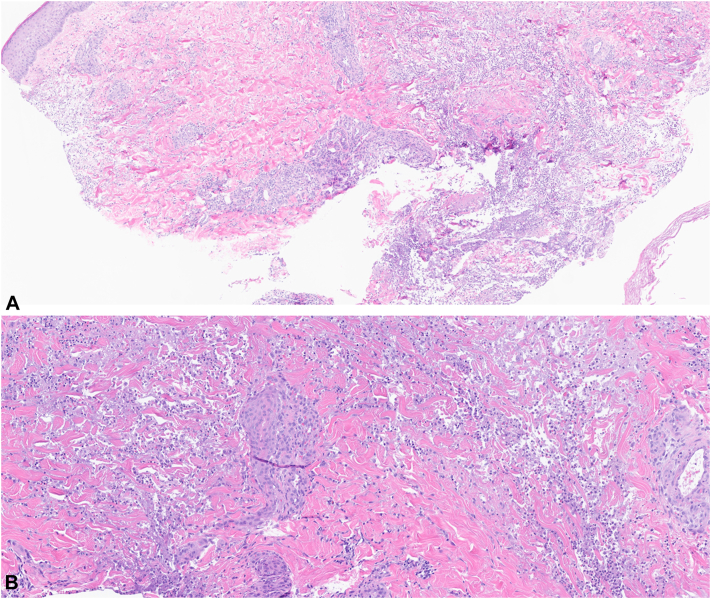
Hematoxylin and eosin stain of biopsy specimen obtained from the lower extremity. (Original magnifications: **A,** ×40; **B,** ×100).

A 26-year-old female with a medical history of bilateral pleural effusions and falciform ligament necrosis was referred to dermatology for a 4-year history of recurrent sterile abscesses primarily involving the lower extremities (Fig 3). The lesions were often preceded by trauma and resulted in abscesses associated with hemorrhagic crusting and purulent drainage. Previous outside biopsies showed dermal inflammation with focal micro-abscess formation, negative for organisms on GMS and gram stain. Prior treatments included intermittent courses of oral antibiotics with no apparent improvement. Repeat biopsy performed at the time of visit showed diffuse neutrophilic inflammation with foamy macrophages and fat necrosis, negative for microorganisms, while evaluation by serum protein electrophoresis demonstrated the absence of an alpha-1 band (Fig 4, *A* and *B*). Further screening revealed a significantly low alpha-1 antitrypsin level of 26 and a homozygous ZZ mutation, indicative of alpha-1 antitrypsin deficiency. The patient was started on doxycycline 100 mg twice a day, and experienced significant improvement of cutaneous lesions following initiation of treatment.

## Discussion

AATD is a hereditary condition caused by mutations in the *SERPINA1* gene (14q32.1), resulting in misfolding of the serine-protease inhibitor AAT. Roughly 90% of people are homozygous for the normal Pi∗M allele (MM). The most common deficiency variant is Pi∗S and the most severe is Pi∗Z. With codominant inheritance, AAT levels drop progressively as mutated alleles accumulate. Accumulation of misfolded AAT in hepatocytes predisposes individuals to liver cirrhosis, while dysregulation of proteases such as elastase by low circulating blood AAT can cause lung damage, progressing to emphysema and chronic obstructive pulmonary disease.^1^^,^^2^ Panniculitis is a rare but serious manifestation of AATD, most frequently diagnosed in the homozygous PiZZ variant. It is sometimes preceded by trauma; however, it often presents spontaneously. While the pathogenesis of the disease is not entirely clear, antiprotease insufficiency along with increased inflammation and neutrophil chemoattraction by Z-ATT polymers serve as an explanation.^2^ Clinical presentation is characterized by the presence of tender, nodular, erythematous skin lesions which may ulcerate and secrete an oily yellow discharge, often found on the trunk and extremities. Histological features display lobular neutrophilic panniculitis followed by necrosis and destruction of fat lobules. Dissolution of dermal collagen with liquefactive necrosis and separation of fat lobules from adjacent septa is common.^3^ Confirmatory diagnosis for AATD is achieved via AAT genotyping/phenotyping following a low (<100 mg/dL) serum result.

These 2 cases demonstrate the importance of a multifaceted diagnostic approach when suspecting AATD as an underlying cause of panniculitis. When subcutaneous tissue is not adequately sampled, as in Case 2, a dense diffuse neutrophilic infiltrate within the dermis may still support a diagnosis of AATD in the appropriate clinical context; serum protein electrophoresis demonstrating absence of the alpha-1 band can be especially helpful in aiding diagnosis. Many of these patients have monoclonal gammopathy and c-ANCA testing as part of their panniculitis work up.^4^ Previous reports have shown AAT1 is the most abundant serum antiprotease produced in hepatocytes and encoded by SERPINA1 (Pi). Because of its abundance, a lower-than-average AAT1 concentration can be represented by a diminished band in alpha-1 on serum protein electrophoresis.^5^ A recent study on the utility of serum electrophoresis in the detection of AAT deficiency reports 91% of patients with diminished alpha-1 bands and low serum AAT were found to have mutations in *SERPINA1*.^6^ Both our cases demonstrated similar findings, prompting further workup for AATD.

An established association exists between ANCA vasculitis and the Pi∗Z allele of the SERPINA1 gene.^7^ AAT is an inhibitor of proteinase-3 (PR3) and previous studies have reported a higher incidence of anti-PR3 antibodies in individuals with AAT phenotypic variants resulting in dysfunctional protein or reduced serum levels.^8^ In our first case, the patient exhibited a markedly positive c-ANCA without clinical or histopathologic evidence of vasculitis. This underscores the importance of interpreting c-ANCA positivity within the appropriate clinical context, as illustrated by our case, while remaining mindful of the established association and physiologic relationship between AAT and PR3.

In conclusion, these cases demonstrate the utility of serum protein electrophoresis studies in the detection of AATD as a potential underlying cause of panniculitis. Given the systemic effects of AATD on the pulmonary and hepatic systems, as well as the challenges in managing AATD-associated panniculitis, prompt recognition and intervention are essential to reduce the risk of serious long-term complications.

## Conflicts of interest

Dr Alavi serves on the board of HS foundation and as a consultant for Abbvie, Almirall, BI, Incyte, InflaRX, Leo, Sanofi, Novartis, UCB, and as investigator for BI and Processa. Author Dale and Drs Jing, Camilleri, and Warrington have no conflicts of interest to declare.
